# The clinical significance of preoperative plasma fibrinogen levels and platelet counts in resectable colon cancer

**DOI:** 10.1186/s12957-021-02180-y

**Published:** 2021-03-11

**Authors:** Berrin Papila Kundaktepe, Cigdem Papila

**Affiliations:** 1 Department of General Surgery, Cerrahpasa Faculty of Medicine, Istanbul University-Cerrahpasa, Istanbul, Turkey; 2 Department of Internal Medicine, Cerrahpasa Faculty of Medicine, Istanbul University-Cerrahpasa, Istanbul, Turkey

**Keywords:** Colon cancer, Platelet counts, Fibrinogen, Lymph node, Venous invasion

## Abstract

**Background and aim:**

Several aspects of the correlation between colon cancer and hemostatic markers are still unknown to many researchers in the field. In this study, we evaluated the association, if any, of preoperative platelet (PLT) counts and plasma fibrinogen levels with postoperative lymph node involvement and venous invasion in colon cancer patients.

**Methods:**

This study retrospectively included eighty patients with colon cancer (mean age 58.09 years; 37% female 63% male).

**Results:**

Patients with negative lymph nodes and venous invasion showed a significantly lower PLT count and higher fibrinogen level than their counterparts, i.e., patients with positive lymph nodes (*p*<0.001, all of them) and venous invasion (*p*<0.001, all of them). The results also showed a positive association of PLT counts and fibrinogen levels with lymphatic invasion (*r*=0.670, *p*<0.001 and *r*=0.639, *p*<0.001, respectively) and a positive association of PLT counts and fibrinogen levels with venous invasion (*r*=0.3988, *p*<0.001 and *r*=0.5268, *p*<0.001, respectively). According to the results of the ROC curve analysis, when the PLT count cutoff was 290/mm^3^, the sensitivity and specificity were 82% and 86.67%, respectively (AUC = 0.8840, *p*<0.0001, 95% CI 0.8084–0.9596). When the fibrinogen level cutoff was 310.0 mg/dL, the sensitivity and specificity were 72% and 96.67%, respectively (AUC 0.8790, *p* <0.0001, 95% CI 0.8067–0.9513).

**Conclusion:**

The preoperative PLT count and plasma fibrinogen level may be considered key markers to monitor postoperative lymph node involvement and venous invasion in colon cancer patients.

## Introduction

Thrombocytosis and activation of the coagulation system are key prognostic factors in various types of malignancies, including colon cancer [1, 2]. In hemostasis and thrombosis, the function of blood platelets (PLTs) can be demonstrated as a temporal sequence of events that involves adhesion, activation, and aggregation to a damaged vascular surface. It is imperative to note that von Willebrand factor (vWF) and fibrinogen are the major ligands that support adhesion and aggregation [3].

In tumor metastasis, activated PLTs play a wide range of roles, such as facilitating tumor cell epithelial-mesenchymal transition (EMT) and degrading the surrounding extracellular matrix (ECM). They also improve vascular permeability and help establish malignancies in distant tissues. Furthermore, PLTs facilitate tumor invasion and metastasis by intensifying tumor cell-associated tissue factor expression, a key determinant of the coagulation cascade [4].

The major ligand of integrin αIIbβ3 (GP IIb-IIIa) is one of the most abundant PLT surface receptors that influence the implication of fibrinogen in tumor metastasis [5]. Moreover, fibrinogen is necessary for the well-characterized paradigm of hemostasis and thrombosis, owing to its role as a central ligand that supports interactions between PLTs. It also acts as an important cleavage substrate for thrombin in coagulation [6]. In tumor biology research, fibrinogen is known to support PLT-fibrinogen tumor embolism with the invasion of tumor cells [7]. Research studies have also found evidence that increased levels of fibrinogen are associated with a high risk of colon cancer development [8].

The current retrospective study aimed to evaluate the association, if any, of preoperative PLT counts and fibrinogen levels with postoperative lymph node involvement and venous invasion in patients with colon cancer.

## Materials and methods

The study protocol received approval from the Ethics Committee of Cerrahpaşa Medical Faculty (No.: 8304 5809- 604.01.02). This retrospective study was completed from January 2019 to February 2020 at the Department of Medical Oncology, Istanbul University-Cerrahpaşa, Cerrahpaşa Medical Faculty. It included 80 patients (mean age 58.09 years; 370% female, 6350% male) with colon cancer. The study participants were of Turkish descent and had no distant organ metastasis.

### Patient characteristics

1. Patients with postoperative metastatic lymph nodes were divided into 3 groups: those with zero lymph nodes, less than 4 lymph nodes, and 4 and more than 4 lymph nodes. In total, 9 patients had zero lymph nodes, 38 patients had fewer than 4 metastatic lymph nodes, and 33 patients had 4 or more lymph nodes.
2. The histological grades were as follows: low grade in 48 patients and high grade in 32 patients.
3. T (tumor) status:
  - T1 (tumor limited to the submucosa): 9 patients
  - T2 (tumor invaded the muscularis propria layer): 41 patients
  - T3 (tumor invaded the pericolorectal fatty tissue and subserosa layer): 30 patients
4. Tumor size (one group, less than 4 cm; other groups, 4 cm and above): 37 patients had tumors < 4 cm and 43 patients had tumors ≥ 4 cm.

Patient medical records were used to collect data, including age, sex, and medical history, along with hemoglobin (Hb), leukocyte (WBC), and PLT counts and fibrinogen levels in the preoperative routine blood examination. Additional data collected for the current study included postoperative histopathological reports for tumors, lymph node involvement (negative or positive), and venous invasion (negative or positive). In this study, potential curative surgery for patients was defined. None of the study participants had received preoperative chemotherapy, radiotherapy, or immunotherapy.

#### Inclusion criteria

The following are the inclusion criteria: patients who underwent operations for T1, T2, and T3 colon tumors; those without distant organ metastases; those who did not receive anticoagulant and anti-PLT therapy; those who did not have liver, hematologic, rheumatologic, cardiac, and renal diseases; and those who are smokers.

#### Exclusion criteria

The following are the exclusion criteria: the patients included in the study were between the ages of 45–70 years, and the mean patient age was 58 years; patients in the higher age group had cardiac complaints, hypertension, and diabetes, and anticoagulants were used; these patients were excluded from the study because their fibrinogen and PLT levels and functions were adversely affected; patients with distant metastases were also excluded. The liver is the “most frequently metastasized organ” of colon cancer. Patients with liver metastases were excluded because these metastases also affect the bleeding coagulation mechanism and fibrinogen and PLT levels.

Colon cancer patients who did not have complete follow-up data or with active concurrent infection were excluded. Additionally, it was confirmed that there was no distant organ metastasis in any of the study participants. Patients with a hematologic disease or other infections that could influence the level of fibrinogen, including liver disease, kidney disease, myocardial infarction, and hypertension, were also excluded. Other factors, such as the use of procoagulant or anticoagulant drugs and a history of blood transfusion, were not taken into account in this study. The results of physical tests and routine laboratory tests along with diagnostic imaging (computed tomography and positron emission tomography), biopsies, and postoperative pathology reports were used to evaluate the study participants.

Blood samples, in general, were obtained prior to surgery. An automatic hematology analyzer (Beckman Coulter, Brea, CA, USA) was used to obtain samples that were collected in standardized tubes containing dipotassium ethylenedinitrilotetraacetic acid (EDTA) for complete blood count (CBC) parameters [Hb (gr/dL), WBC (× 10^9^/L), and PLT (/mm^3^)]. The Clauss method, using a Fibrintimer II coagulometer and Multifibren U Kit (Siemens Healthcare Diagnostics, Germany), was used to measure plasma fibrinogen. As mentioned earlier, we used patients’ medical records to obtain their PLT counts in preoperative routine blood examinations. The manufacturer recommended normal ranges for plasma fibrinogen concentrations of 180–350 mg/dL and PLT counts of 156–348/mm^3^.

### Statistical analysis

SPSS 20.0 (SPSS Inc., Chicago, IL, USA) was used for statistical analyses. First, all patients’ information was checked for normality. Normally distributed continuous variables are shown as the mean ± standard deviation (SD). One-way analysis of variance (ANOVA) followed by Tukey’s multiple comparison tests was used to evaluate the variables. Pearson’s correlation was used to analyze the numerical data, while Spearman’s correlation was employed to analyze the nominal data. Receiver operating characteristic (ROC) curve analysis was used to determine the separation power of the parameters. As a result of the ROC analysis, cutoff values were determined by using the Youden index. To determine the risk of having values above the cutoff value, risk analysis was performed, and odds ratios (ORs) were obtained. Since small numbers increase estimation bias, Haldane’s correction was used. *p* values < 0.05 were considered statistically significant.

## Results

Table 1 presents the different characteristics of colon cancer patients. Table 2 shows the mean and standard deviations of the patients’ clinicopathologic parameters. The current study compared lymph nodes (negative and positive) and venous invasion (negative and positive) in colon cancer patients post-operation. Patients with negative lymph nodes and venous invasion had significantly lower PLT counts and fibrinogen levels than patients with positive lymph nodes (*p*<0.001, all of them) and venous invasion (*p*<0.001, all of them) (Table 2).

**Table 1:** Characteristics of patients with colon cancer

Characteristic	*n* (%)
Total number of patients	80
Sex	
Female	30 (37)
Male	50 (63)
Age (years)	58.09 ± 6.06
BMI (kg/m^2^)	27.41±2.95
Hemoglobin (g/dL)	10.50±0.80
Leukocytes (×10^9^/L)	6.79±1.44
Platelet counts (/mm^3^)	309.5±85.2
Fibrinogen (mg/dL)	316.00±97.68

*BMI* body mass index

**Table 2:** Clinicopathological parameters of colon cancer patients without distant metastasis

Characteristic	Venous invasion status		***p***	***t***; df	95% confidence interval
	Negativity (***n***=38)	Positivity (***n***=42)			
Age (years)	58.47 ± 6.43	57.74 ± 5.76	0.591	*t*=0.540; df=78	− 3.449 to 1.977
BMI (kg/m^2^)	27.15 ± 2.81	27.65 ± 3.08	0.454	*t*=0.7524; df=78	− 0.819 to 1.814
Platelet counts (/mm^3^)	269.3 ± 79.18	344.3 ± 93.79a*	<0.001	*t*=3.840; df=78	36.09 to 113.8
Fibrinogen (mg/dL)	258.8 ± 69.01	345.8 ± 72.75a*	<0.001	*t*=5.474; df=78	55.37 to 118.7
**Characteristic**	**Lymph invasion status**		***p***	***t*; df**	**95% confidence interval**
	**Negativity (*n*=30)**	**Positivity (*n*=50)**			
Age (years)	57.97±6.96	58.16±5.52	0.891	*t*=0.137; df=78	− 2.610 to 2.997
BMI (kg/m^2^)	27.55 ±2.40	27.32±3.25	0.737	*t*=0.3375; df=78	− 1.593 to 1.131
Platelet counts (/mm^3^)	227.5±52.65	357.4±79.27a*	<0.001	*t*=7.972; df=78	97.46 to 162.3
Fibrinogen (mg/dL)	236.5±49.81	345.3±71.48a*	<0.001	*t*=7.329; df=78	79.24 to 138.4

*BMI* body mass index

*p<0.001

Table 3 shows the strength of fibrinogen level and PLT count values in separating venous invasion positivity. According to the results of the ROC curve analysis, when the PLT count cutoff was 330/mm^3^, the sensitivity and specificity were 66.67% and 81.58%, respectively (AUC = 0.716, *p* = 0.0009, 95% CI 0.599–0.833). When the fibrinogen level cutoff was 310.0 mg/dL, the sensitivity and specificity were 71.43% and 81.58%, respectively (AUC = 0.8055, *p* <0.0001, 95% CI 0.710–0.901). If the PLT count and fibrinogen level were above the cutoff values, the likelihood of venous invasion being positive increased by 8.857 and 7 times, respectively (Table 4).

**Table 3:** Cutoff, sensitivity, and specificity values for platelet and fibrinogen values indicating venous invasion positivity and lymph node positivity by ROC curve analysis

Variable	AUC	*p*	Asymptotic 95% confidence interval	Cutoff	Sensitivity	Specificity
Venous invasion positivity						
Platelet counts	0.716	<0.001	0.599 to 0.833	> 330	66.67%	81.58%
Fibrinogen	0.805	<0.001	0.710 to 0.901	> 310	71.43%	81.58%
Lymph node positivity						
Platelet counts	0.884	<0.001	0.808 to 0.960	> 290	82%	86.67%
Fibrinogen	0.879	<0.001	0.807 to 0.951	> 3100	72%	96.67%

**Table 4:** Cutoff values and odds ratios for platelet and fibrinogen values indicating venous invasion positivity and lymph node positivity by regression analysis

Variable	Cutoff	Odds ratio	Asymptotic 95% confidence interval	*z* statistic	*p*
Venous invasion positivity					
Platelet counts	> 330	8.86	3.127 to 25.092	4.106	< 0.001
Fibrinogen	> 310	7.00	2.6150 to 18.738	3.873	0.001
Lymph node positivity					
Platelet counts	> 290	29.61	8.264 to 106.097	5.204	< 0.001
Fibrinogen	> 310	74.57	9.252 to 601.042	4.050	0.001

Table 3 shows the strength of fibrinogen level and PLT count values in separating lymph node positivity. According to the results of the ROC curve analysis, when the PLT count cutoff was 290/mm^3^, the sensitivity and specificity were 82% and 86.67%, respectively (AUC = 0.8840, *p*<0.0001, 95% CI 0.8084–0.9596). When the fibrinogen level cutoff was 310.0 mg/dL, the sensitivity and specificity were 72% and 96.67%, respectively (AUC = 0.8790, *p* <0.0001, 95% CI 0.8067–0.9513). If the PLT count and fibrinogen level were above the cutoff values, the likelihood of lymphatic invasion being positive increased by 29.61 and 74.57 times, respectively (Table 4).

Both parameters were found to be statistically significant in differentiating venous invasion and positive lymph nodes. The study results showed a positive association of PLT counts and fibrinogen levels with lymphatic invasion (*r*=0.670, *p*<0.001 and *r*=0.639, *p*<0.001, respectively, Table 5) and a positive association of PLT counts and fibrinogen levels with venous invasion (*r*=0.3988, *p*<0.001 and *r*=0.5268, *p*<0.001, respectively, Table 5).

**Table 5:** Correlation analyses between platelet count, fibrinogen level, and venous invasion and positive lymph nodes

	Lymphatic invasion positivity		Venous invasion positivity	
	r	*p*	r	*p*
Platelet counts	0.670	<0.001	0.399	<0.001
Fibrinogen	0.639	<0.001	0.527	<0.001

## Discussion

Several types of cancer, especially gastric, colorectal, pancreatic, prostate, lung, and esophageal squamous cell carcinomas, have been associated with elevated plasma fibrinogen levels and PLT counts [2, 8–10]. The current study findings showed that patients with negative lymph nodes have significantly lower PLT counts and fibrinogen levels than patients with positive lymph nodes. According to ROC curve analysis, the fibrinogen level and PLT count had high specificity in distinguishing venous invasion positivity, with a sensitivity and specificity of 82% and 86.67%, respectively, when the PLT count cutoff to distinguish lymph node positivity was 290 × 10^4^/μL. The findings also showed that preoperative PLT counts and plasma fibrinogen levels may be used as key markers to monitor postoperative lymph node involvement and venous invasion.

In the last few decades, thrombocytosis has been used to prognosticate reduced chances of survival from solid tumors. Additionally, various studies have evaluated the prognostic significance of thrombocytosis in colon cancer [11–18]. These studies have significantly demonstrated the correlation between thrombocytosis, hyperfibrinogenemia, and malignancy. Sasaki et al. [15] examined the link between thrombocytosis and the survival of 636 colorectal cancer patients. They reported that preoperative thrombocytosis is a potential prognostic factor of poor survival in colorectal cancer patients. Furthermore, several reports have demonstrated the link between thrombocytosis and the survival of patients with colorectal cancer [13, 15]. Lin et al. [2] reported that an elevated PLT count might influence the development of colorectal cancer, and the preoperative PLT count might be a predictive factor of survival in patients with colorectal cancer. Navarro et al. [19] showed the presence of a predictive characteristic of colorectal cancer in the blood that can be measured with an interaction index of various blood parameters, such as PLT counts and levels of hemoglobin, fibrinogen, hematocrit, total leukocytes, eosinophils, and neutrophils. The current study showed that patients with negative lymph nodes and venous invasion had significantly lower PLT counts than patients with positive lymph nodes and venous invasion. The study showed a positive association of PLT counts and fibrinogen levels with lymphatic invasion and a positive association of PLT counts and fibrinogen levels with venous invasion. If the PLT count and fibrinogen level were above the cutoff values, the likelihood of venous invasion being positive increased by 8.857 and 7 times, respectively. A recent study showed a strong association between the PLT count and 5-year overall and cancer-related survival after the stratification of patients as per the TNM staging system. This study validated that PLTs are likely to contribute to the progression of cancer by promoting neoangiogenesis, increasing microvessel permeability and the extravasation of cancer cells, generating growth factors (e.g., VEGF, PDGF, and TGF-β), and promoting the interaction between tumor cells and endothelia at metastatic sites [20]. The preoperative PLT count thus acts as a simple tool to determine the possibility of undergoing incomplete tumor resection [21].

The progression of cancer has been linked to the hemostatic system of the host. The predictive factors of preoperative plasma hemostasis for survival in colon cancer patients remain quite interesting. Thromboembolic complications are considered one of the causes of mortality from cancer or a malignant disease [22, 23]. Son et al. [24] evaluated the prognostic significance of fibrinogen and inflammation-based scores, such as albumin, C-reactive protein, and neutrophil, lymphocyte, and PLT counts, as markers of the inflammatory response in colon cancer. In this study, preoperative hyperfibrinogenemia was found to be a statistically significant prognostic factor for both disease-free survival and overall survival after curative resection of nonmetastatic colon cancer. They also reported that fibrinogen may play an important role as an inflammatory marker and prognostic marker in colon cancer. In contrast, none of the other inflammatory-based scores evaluated was significantly associated with survival. A previous study found a correlation between the development of PLT-fibrin-tumor cell aggregates and endothelial adhesion and potential metastasis [25]. Sun et al. [26] reported a close association of fibrinogen with a wide range of clinicopathological features and 5-year survival rates of patients with colon cancer. The preoperative level of fibrinogen may also be considered a promising prognostic marker for colon cancer in patients receiving radical surgery. Our study found that patients with negative lymph nodes have significantly lower fibrinogen levels than their counterparts with positive lymph nodes. It has also been observed that colon cancer patients with high fibrinogen levels and PLT counts are at an increased tendency to experience clotting. These parameters arise particularly in association with the lymph nodes (+). According to Yamashita et al. [27], hyperfibrinogenemia is clinically relevant in the recurrence of a tumor prior to a systemic inflammatory response. Thus, it can be considered a key prognostic factor of tumor recurrence in the preinflammatory stage of colon cancer. Other results of their study might support the perception that hyperfibrinogenemia can accelerate lymphatic and hematogenous metastases of advanced gastric cancer—key indicators of the prognosis in T2 gastric cancer. Therefore, in a situation with no peritoneal involvement, hyperfibrinogenemia is a key biomarker for predicting possible metastasis and a poor clinical outcome in T2 gastric cancer. They may also provide cancer cells with favorable circumstances to metastasize through the lymphatic system [28]. Additionally, the preoperative level of plasma fibrinogen is considered an important predictor of lymphatic metastasis in intestinal-type gastric cancer [29]. The current study results confirmed the role of plasma fibrinogen as both an inflammatory marker and a prognostic marker in colon cancer [27, 30, 31].

### Strong points of the study

Malignant tumors usually progress rapidly and cause early lymphatic and venous invasion in people who are prone to clotting. As hematological parameters, especially thrombocyte and fibrinogen levels, are closely related to the coagulation process, it has been shown that high preoperative fibrinogen and thrombocyte levels play an important role in lymphatic and venous invasion in colon tumors.

However, the present study had some limitations similar to those of other retrospective studies. The number of patients was very limited. There was no patient group with extrahepatic distant metastases. Associations with disease-free and overall survival could not be evaluated.

## Conclusion

Our results showed an association of increased circulating fibrinogen levels and PLT counts with a high risk of the formation of colon cancer in patients with positive lymph nodes and venous invasion. However, the thrombosis formation mechanism in colon cancer patients has not been clearly explained. The preoperative plasma fibrinogen level and PLT count are considered useful markers after conducting validation studies at other laboratories with colon cancer patients without distant organ metastasis. Further studies using a larger sample size will possibly establish the roles of thrombocytosis and hyperfibrinogenemia in patients with colon cancer.

## Data Availability

The data sets analyzed during the current study are available from the corresponding author on reasonable request.

## Abbreviations

PLT: Platelet
EMT: Epithelial-mesenchymal transition
ECM: Extracellular matrix
vWF: von Willebrand factor
CBC: Complete blood count
ROC: Receiver operating characteristic
Hb: Hemoglobin
WBC: White blood cell

## References

[1] Costantini V, Zacharski LR, Moritz TE, Edwards RL (1990). The platelet count in carcinoma of the lung and colon. Thromb Haemost..

[2] Lin MS, Huang JX, Zhu J, Shen HZ (2012). Elevation of platelet count in patients with colorectal cancer predicts tendency to metastases and poor prognosis. Hepatogastroenterology..

[3] Franco AT, Ware J (2019). Pathophysiology 2: the role of platelets in cancer biology. Cancer Treat Res..

[4] Meikle CK, Kelly CA, Garg P, Wuescher LM, Ali RA, Worth RG (2017). Cancer and thrombosis: the platelet perspective. Front Cell Dev Biol..

[5] Lavergne M, Janus-Bell E, Schaff M, Gachet C, Mangin PH (2017). Platelet integrins in tumor metastasis: do they represent a therapeutic target?. Cancers (Basel).

[6] Chapin JC, Hajjar KA (2015). Fibrinolysis and the control of blood coagulation. Blood Rev..

[7] Jain S, Harris J, Ware J (2010). Platelets: linking hemostasis and cancer. Arterioscler Thromb Vasc Biol..

[8] Erdogan S, Yilmaz FM, Yazici O, Yozgat A, Sezer S, Ozdemir N, Uysal S, Purnak T, Sendur MA, Ozaslan E (2016). Inflammation and chemerin in colorectal cancer. Tumour Biol..

[9] Wang J, Liu H, Shao N, Tan B, Song Q, Jia Y, Cheng Y (2015). The clinical significance of preoperative plasma fibrinogen level and platelet count in resectable esophageal squamous cell carcinoma. World J Surg Oncol..

[10] Sylman JL, Boyce HB, Mitrugno A, Tormoen GW, Thomas IC, Wagner TH, Lee JS, Leppert JT, McCarty OJT, Mallick P (2018). A temporal examination of platelet counts as a predictor of prognosis in lung, prostate, and colon cancer patients. Sci Rep..

[11] Ishizuka M, Nagata H, Takagi K, Iwasaki Y, Kubota K (2012). Preoperative thrombocytosis is associated with survival after surgery for colorectal cancer. J Surg Oncol.

[12] Cravioto-Villanueva A, Luna-Perez P, Gutierrez-de la Barrera M, Martinez-Gómez H, Maffuz A, Rojas-Garcia P, Perez-Alvarez C, Rodriguez-Ramirez S, Rodriguez-Antezana E, Ramirez-Ramirez L (2012). Thrombocytosis as a predictor of distant recurrence in patients with rectal cancer. Arch Med Res.

[13] Kandemir EG, Mayadagli A, Karagoz B, Bilgi O, Turken O, Yaylaci M (2005). Prognostic significance of thrombocytosis in node-negative colon cancer. J Int Med Res.

[14] Baranyai Z, Krzystanek M, Josa V, Dede K, Agoston E, Szász AM, Sinkó D, Szarvas V, Salamon F, Eklund AC (2014). The comparison of thrombocytosis and platelet-lymphocyte ratio as potential prognostic markers in colorectal cancer. Thromb Haemost.

[15] Sasaki K, Kawai K, Tsuno NH, Sunami E, Kitayama J (2012). Impact of preoperative thrombocytosis on the survival of patients with primary colorectal cancer. World J Surg.

[16] Kim HJ, Choi G, Park JS, Park S, Kawai K, Watanabe T (2015). Clinical significance of thrombocytosis before preoperative chemoradiotherapy in rectal cancer: predicting pathologic tumor response and oncologic outcome. Ann Surg Oncol.

[17] Wan S, Lai Y, Myers RE, Li B, Hyslop T, London J (2013). Preoperative platelet count associates with survival and distant metastasis in surgically resected colorectal cancer patients. J Gastrointest Cancer.

[18] Nyasavajjala SM, Runau F, Datta S, Annette H, Shaw AG, Lund JN (2010). Is there a role for pre-operative thrombocytosis in the management of colorectal cancer?. Int J Surg.

[19] Navarro Rodríguez JM, Gallego Plazas J, Borrás Rocher F, Calpena Rico R, Ruiz Macia JA, Morcillo Ródenas MÁ (2017). Is it possible to predict the presence of colorectal cancer in a blood test? A probabilistic approach method. Rev Esp Enferm Dig..

[20] Pedrazzani C, Mantovani G, Fernandes E, Bagante F, Luca Salvagno G, Surci N (2017). Assessment of neutrophil-to-lymphocyte ratio, platelet-to-lymphocyte ratio and platelet count as predictors of long-term outcome after R0 resection for colorectal cancer. Sci Rep..

[21] Pedrazzani C, Turri G, Mantovani G, Conti C, Ziello R, Conci S, Campagnaro T, Ruzzenente A, Guglielmi A (2019). Prognostic value of thrombocytosis in patients undergoing surgery for colorectal cancer with synchronous liver metastases. Clin Transl Oncol..

[22] Sallah S, Ahmad O, Kaiser HE (2000). Pathogenesis of thrombotic disorders in patient with cancer. Cancer.

[23] Alatri A, Carnovali M, Prandoni P (2000). Venous thromboembolism and neoplasms. Ann Ital Med Int.

[24] Son HJ, Park JW, Chang HJ, Kim DY, Kim BC, Kim SY, Park SC, Choi HS, Oh JH (2013). Preoperative plasma hyperfibrinogenemia is predictive of poor prognosis in patients with nonmetastatic colon cancer. Ann Surg Oncol..

[25] Gay LJ, Felding-Habermann B (2011). Contribution of platelets to tumour metastasis. Nat Rev Cancer..

[26] Sun ZQ, Han XN, Wang HJ, Tang Y, Zhao ZL, Qu YL, Xu RW, Liu YY, Yu XB (2014). Prognostic significance of preoperative fibrinogen in patients with colon cancer. World J Gastroenterol..

[27] Yamashita H, Kitayama J, Taguri M, Nagawa H (2009). Effect of preoperative hyperfibrinogenemia on recurrence of colorectal cancer without a systemic inflammatory response. World J Surg..

[28] Yamashita H, Kitayama J, Kanno N, Yatomi Y, Nagawa H (2006). Hyperfibrinogenemia is associated with lymphatic as well as hematogenous metastasis and worse clinical outcome in T2 gastric cancer. BMC Cancer..

[29] Yamashita H, Kitayama J, Nagawa H (2005). Hyperfibrinogenemia is a useful predictor for lymphatic metastasis in human gastric cancer. Jpn J Clin Oncol..

[30] Tang L, Liu K, Wang J, Wang C, Zhao P, Liu J (2010). High preoperative plasma fibrinogen levels are associated with distant metastases and impaired prognosis after curative resection in patients with colorectal cancer. J Surg Oncol..

[31] Kawai K, Kitayama J, Tsuno NH, Sunami E, Nagawa H (2011). Hyperfibrinogenemia after preoperative chemoradiotherapy predicts poor response and poor prognosis in rectal cancer. Int J Colorectal Dis..

